# Reviewed article: Araujo MC, Passinho RS, Pereira RSF, Mora DJ. Temporal trends in incidence and mortality from pulmonary tuberculosis: time series study, Southern Bahia, Brazil, 2010-2023. Epidemiol Serv Saude. 2025:34;e20240778.

**DOI:** 10.1590/S2237-96222025v34e20240778.b

**Published:** 2025-08-04

**Authors:** Cássia CP Mendicino

**Affiliations:** Secretaria de Estado de Saude de Minas Gerais

**Table:** 

Rating	Excellent	Good	Average	Below average	Poor
Interest		✓			
Quality		✓			
Originality			✓		
Overall		✓			

**Table:** 

Questionnaire	Yes	No	Not applicable
Does the manuscript contain new and significant information to justify publication?	✓		
Does the Abstract (Summary) clearly and accurately describe the content of the article?	✓		
Is the problem significant and concisely stated?	✓		
Are the methods described comprehensively?	✓		
Are the interpretations and conclusions justified by the results?	✓		
Is adequate reference made to other work in the field?	✓		

## Manuscript Structure

Length of article is: Adequate

Number of tables is: Too few

Number of figures is: Too few

Please state any conflict(s) of interest that you have in relation to the review of this paper (state “none” if this is not applicable): None

## Comments

Nothing to declare.

